# The impact of sample size on the reproducibility of voxel-based lesion-deficit mappings

**DOI:** 10.1016/j.neuropsychologia.2018.03.014

**Published:** 2018-07-01

**Authors:** Diego L. Lorca-Puls, Andrea Gajardo-Vidal, Jitrachote White, Mohamed L. Seghier, Alexander P. Leff, David W. Green, Jenny T. Crinion, Philipp Ludersdorfer, Thomas M.H. Hope, Howard Bowman, Cathy J. Price

**Affiliations:** a Wellcome Centre for Human Neuroimaging, Institute of Neurology, University College London, London WC1N 3BG, United Kingdom; b Department of Speech, Language and Hearing Sciences, Faculty of Medicine, Universidad de Concepcion, PO Box 160-C, Concepcion, Chile; c Department of Speech, Language and Hearing Sciences, Faculty of Health Sciences, Universidad del Desarrollo, 4070001 Concepcion, Chile; d Cognitive Neuroimaging Unit, Emirates College for Advanced Education, PO Box 126662, Abu Dhabi, United Arab Emirates; e Institute of Cognitive Neuroscience, Division of Psychology and Language Sciences, University College London, London WC1N 3AR, United Kingdom; f Department of Brain Repair and Rehabilitation, Institute of Neurology, University College London, London WC1N 3BG, United Kingdom; g Department of Experimental Psychology, Division of Psychology and Language Sciences, University College London, London WC1H 0AP, United Kingdom; h Centre for Cognitive Neuroscience and Cognitive Systems and the School of Computing, University of Kent, Canterbury CT2 7NF, United Kingdom; i School of Psychology, University of Birmingham, Birmingham B15 2TT, United Kingdom

**Keywords:** Voxel-based, Lesion-symptom, Lesion, Deficit, Reproducibility, Stroke, Speech production

## Abstract

This study investigated how sample size affects the reproducibility of findings from univariate voxel-based lesion-deficit analyses (e.g., voxel-based lesion-symptom mapping and voxel-based morphometry). Our effect of interest was the strength of the mapping between brain damage and speech articulation difficulties, as measured in terms of the proportion of variance explained. First, we identified a region of interest by searching on a voxel-by-voxel basis for brain areas where greater lesion load was associated with poorer speech articulation using a large sample of 360 right-handed English-speaking stroke survivors. We then randomly drew thousands of bootstrap samples from this data set that included either 30, 60, 90, 120, 180, or 360 patients. For each resample, we recorded effect size estimates and *p* values after conducting exactly the same lesion-deficit analysis within the previously identified region of interest and holding all procedures constant. The results show (1) how often small effect sizes in a heterogeneous population fail to be detected; (2) how effect size and its statistical significance varies with sample size; (3) how low-powered studies (due to small sample sizes) can greatly over-estimate as well as under-estimate effect sizes; and (4) how large sample sizes (*N* ≥ 90) can yield highly significant *p* values even when effect sizes are so small that they become trivial in practical terms. The implications of these findings for interpreting the results from univariate voxel-based lesion-deficit analyses are discussed.

## Highlights

• The same lesion-deficit analysis was repeated on thousands of bootstrap samples.
• Replicability of the original effect was contingent upon the size of the sample.
• With smaller samples, only inflated effect size estimates reached significance.
• With larger samples, even trivial effect sizes yielded significant *p* values.

## 1 Introduction

There is a great deal of evidence showing how both false positive and false negative results increase as sample size decreases (Bakker et al., 2012, Button et al., 2013a, Chen et al., 2018, Cremers et al., 2017, Ingre, 2013, Ioannidis, 2008) and how inadequate statistical power can lead to replication failures (Anderson et al., 2017, Bakker et al., 2012, Perugini et al., 2014, Simonsohn et al., 2014a, Szucs and Ioannidis, 2017). However, the impact of sample size on false negative and false positive rates has never been quantified in mass-univariate voxel-based lesion-deficit mapping (e.g., voxel-based lesion-symptom mapping and voxel-based morphometry). Using data from a large sample of stroke patients, we firstly estimated the magnitude of a lesion-deficit mapping of interest and then formally investigated how effect size and its statistical significance varies with sample size. In addition to demonstrating how small samples can result in over- and under-estimations of effect size, we also highlight an issue with large sample sizes whereby high statistical power dramatically increases the likelihood of detecting effects that are so small that they become uninteresting from a scientific viewpoint (i.e. the fallacy of classical inference; Friston et al., 2012). In other words, statistically significant findings when sample sizes are large can hide the fact that the effect under investigation might be of little importance in practical terms, or, even worse, the result of random chance alone and thereby a false positive (Smith and Nichols, 2018).

To investigate the effect of sample size on the results of univariate voxel-based lesion-deficit mapping, we randomly drew thousands of resamples (with a range of sample sizes) from a set of data from 360 stroke survivors who had collectively acquired a wide range of left hemisphere lesions and cognitive impairments. By using a single patient population and holding all procedures and analyses constant, we ensured that variability in the results across thousands of random resamples cannot be explained by methodological confounds - such as the use of dissimilar recruitment strategies and/or behavioural assessments - that are likely to influence the findings of studies that aggregate data from multiple independent sources (e.g., meta-analyses; Müller et al., 2018). Furthermore, by performing our statistical analyses on actual data, rather than running simulations on synthetically-generated data, we attempt to recreate real-world scenarios that could be encountered by researchers conducting lesion-deficit mapping studies.

The goal of our resampling procedure was to estimate the degree to which the magnitude and statistical significance of the exact same lesion-deficit mapping (i.e. brain areas where damage is associated with difficulties articulating speech) changed with sample size. We report the frequency of significant and non-significant effects (using standard significance thresholds) for 6 different sample sizes: *N* = 30, 60, 90, 120, 180 and 360. In a real world situation where only one sample is typically analysed, results are far more likely to be published when they reach statistical significance (i.e. the associated *p* values are below a certain alpha threshold) than when they fail to produce any evidence in favour of the tested hypothesis. This is known as “publication bias” (e.g., Fusar-Poli et al., 2014; Ioannidis et al., 2014; Johnson et al., 2017; Simonsohn et al., 2014a). For example, the prevalence of “positive” (i.e. statistically significant) findings across a wide range of publication outlets, including neuroscience and psychology, has been shown to be well over 80% (Fanelli, 2010, Fanelli, 2012), which suggests that the vast majority of studies that yield “negative” findings are left unpublished. This is known as “the file drawer problem” (Franco et al., 2014, Simonsohn et al., 2014b). Moreover, the number of “positive” results in the fMRI (David et al., 2013) and brain volume abnormalities (Ioannidis, 2011) literature has been demonstrated to be significantly greater than the number expected on the basis of statistical power considerations.

By leaving non-significant results in the file drawer, it becomes increasingly difficult to ascertain which effects are true (and would replicate in subsequent studies) and which are false (and would not replicate in subsequent studies). A highly significant result from a heterogeneous population could, for example, be driven by random noise when a study selects, by chance, a sample that renders an inflated (unstandardized) effect size and under-estimated variance. In line with this rationale, it has been claimed that more than 50% of all significant effects reported in cognitive neuroscience and psychology journals are likely to correspond to false positives (Szucs and Ioannidis, 2017).

Our study therefore speaks directly to the “replication crisis” that is currently being highlighted in psychology and neuroscience (Forstmeier et al., 2017, Gelman and Geurts, 2017, Ioannidis, 2005, Loken and Gelman, 2017, Munafò et al., 2017, Pashler and Wagenmakers, 2012). In the field of psychology, for example, a large-scale collaborative initiative reported that it could only successfully replicate less than 40% of original effects from a representative set of one hundred randomly selected studies (Open Science Collaboration, 2015). Similar failed replication attempts have also been recorded in other research areas including those investigating structural brain-behaviour correlations (Boekel et al., 2015) and the blood-oxygen-level-dependent response (Chen et al., 2018, Wende et al., 2017).

## 2 Materials and methods

### 2.1 Participants

Data from all participants were retrieved from the Predicting Language Outcome and Recovery After Stroke (PLORAS) database (Price et al., 2010b; Seghier et al., 2016). At a minimum, the data available for each patient included: a full assessment of speech and language abilities and a 3D lesion image, in standard space, created from a T1-weighted high resolution (1 mm isotropic voxels) anatomical whole-brain volume, using our automated lesion identification software (Seghier et al., 2008). The study was approved by the Joint Research Ethics Committee of the National Hospital for Neurology and Neurosurgery and the Institute of Neurology. All patients gave written informed consent prior to participation and were compensated for their time.

Our patient selection criteria included all adult stroke survivors who: (i) had a left-hemisphere lesion (as attested by a clinical neurologist: co-author A.P.L.) that was greater than 1 cm^3^ (as measured by our automated lesion identification tool; Seghier et al., 2008); (ii) had no history of neurological or psychiatric illness that was not related to their stroke; (iii) were right-handed (pre-morbidly); and, (iv) were native speakers of English. Additionally, individuals who had missing scores on the tasks of interest (see below for details) were excluded from the study. These criteria were met by a total of 363 stroke patients whose data were collected between April 2003 and December 2016. To ensure that our full sample could be divided evenly into smaller resampled data sets (see below for details), we additionally excluded from any further analyses the 3 patients with the smallest lesions (i.e. 1.2, 1.3 and 1.4 cm^3^ in size). See Table 1 for demographic and clinical details of the full sample of 360 stroke patients.

**Table 1:** Summary of demographic and clinical data for full sample.

**Factor**		***N*****= 360**
Age at stroke	*M*	54.4
onset (years)	*SD*	12.9
	Range	17.2–86.5
Age at testing	*M*	59.4
(years)	*SD*	12.4
	Range	21.3–90.0
Time post-stroke	*M*	4.9
(years)	*SD*	5.2
	Range	0.2–36.0
Education	*M*	14.5
(years)*	*SD*	3.2
	Range	10.0–30.0
Lesion size	*M*	85.7
(cm^3^)	*SD*	87.6
	Range	1.5–386.2
Gender	Males	250
	Females	110
Rep-N	Imp/Non	132/228
	*M*	54.4
	*SD*	9.1
Writt-PN	Imp/Non	105/255
	*M*	58.6
	*SD*	8.7
Recog-M	Imp/Non	37/323
	*M*	53.9
	*SD*	7.0
Sem-A	Imp/Non	36/324
	*M*	56.6
	*SD*	6.1
A_W_-P	Imp/Non	77/283
	*M*	57.0
	*SD*	6.8

Imp/Non = number of patients with impaired/non-impaired performance.

* Missing data: three patients.

### 2.2 Behavioural assessment

All patients recruited to the PLORAS database are assessed on the Comprehensive Aphasia Test (CAT) (Swinburn et al., 2004). The CAT is a fully standardised test battery, which consists of a total of 27 different tasks. For ease of comparison across tasks, the authors of the CAT encourage the conversion (through a non-linear transformation) of raw scores into T-scores, which represent how well the patient performed relative to a reference population of 113 patients with aphasia, 56 of whom were tested more than once. For example, a T-score of 50 indicates the mean of the patient sample used to standardise the CAT, whereas a T-score of 60 represents one standard deviation above the mean. Most people without post-stroke aphasia would therefore be expected to score above the average of the patient standardisation sample on any given task from the CAT. The threshold for impairment is defined relative to a second reference population of 27 neurologically-normal controls. Specifically, it is the point below which the score would place the patient in the bottom 5% of the control population (Swinburn et al., 2004). Lower scores indicate poorer performance. Importantly, the two standardisation samples referred to before (i.e. 113 patients with aphasia and 27 neurologically-normal controls) are completely independent of the data we report in the current paper (for more details on the standardisation samples, see Swinburn et al., 2004).

As stated in the CAT manual (p. 71), the main advantages of converting raw scores into T-scores is that this allows: (i) scores from different tasks to be compared because they have been put on a common scale; and (ii) the use of parametric statistics given that T-scores are normally distributed scores with a mean of 50 and a standard deviation of 10.

The current study focused exclusively on a total of 5 tasks from the CAT. Task 1 used nonword repetition to assess the patient's ability to articulate speech. Task 2 used written picture naming to test the patient's ability to find the names of objects (lexical/phonological retrieval). Tasks 3–5 tested the patient's ability to recognise, process and remember the semantic content of pictures and auditory words. Task details were as follows:

#### 2.2.1 Task 1

The CAT nonword repetition (Rep-N) task aurally presents five nonsense words (e.g., gart), one at a time, with instructions to repeat them aloud. Immediate correct responses were given a score of 2; incorrect responses were given a score of 0; correct responses after a self-correction or a delay (> 5 s) were given a score of 1. Articulatory errors (e.g., dysarthric distortions) not affecting the perceptual identity of the target were scored as correct. Verbal, phonemic, neologistic and apraxic errors were scored as incorrect. T-scores equal to or below 51 constitute the impaired range.

#### 2.2.2 Task 2

The CAT written picture naming (Writt-PN) task visually presents five pictures of objects (e.g., tank), one at a time, with instructions to write their names down. Letters in the correct position were given a score of 1 each. Substitutions, omissions and transpositions were given a score of 0. One point was deducted from the total score if one or more letters were added to the target word. T-scores equal to or below 54 constitute the impaired range.

#### 2.2.3 Task 3

The CAT semantic associations (Sem-A) task visually presents five pictures of objects simultaneously. The instructions were to match the picture at the centre (e.g., mitten) with one of four possible alternatives according to the strongest semantic association (e.g., hand, sock, jersey, and lighthouse). The inclusion of a semantically related distractor (e.g., sock) encouraged deeper levels of semantic processing/control. There are a total of ten test trials plus a practice one at the beginning. Correct responses were given a score of 1; incorrect responses were given a score of 0. T-scores equal to or below 47 constitute the impaired range.

#### 2.2.4 Task 4

The CAT recognition memory (Recog-M) task visually presents each of the ten central items from the CAT semantic associations task (one at a time) along with three unrelated distractors. The instructions were to indicate which of the four pictures on display had been seen before. There are a total of ten test trials plus a practice one at the beginning. The scoring system for this task was identical to that used in the semantic associations task. T-scores equal to or below 43 constitute the impaired range.

#### 2.2.5 Task 5

The CAT auditory word-to-picture matching (A_W_-P) task involves hearing a word produced by the examiner and selecting the picture among four possible alternatives that best matches the meaning of the heard word. There are a total of fifteen test trials plus a practice one at the beginning. Immediate correct responses were given a score of 2; incorrect responses were given a score of 0; correct responses after a self-correction or a delay (> 5 s) were given a score of 1. T-scores equal to or below 51 constitute the impaired range.

### 2.3 MRI data acquisition, pre-processing and lesion identification

T1-weighted high resolution anatomical whole-brain volumes were available for all patients (*N* = 360). Four different MRI scanners (Siemens Healthcare, Erlangen, Germany) were used to acquire the structural images: 167 patients were imaged on a 3 T Trio scanner, 131 on a 1.5 T Sonata scanner, 57 on a 1.5 T Avanto scanner, and five on a 3 T Allegra scanner. For anatomical images acquired on the 1.5 T Avanto scanner, a 3D magnetization-prepared rapid acquisition gradient-echo (MPRAGE) sequence was used to acquire 176 sagittal slices with a matrix size of 256 × 224, yielding a final spatial resolution of 1 mm isotropic voxels (repetition time/echo time/inversion time = 2730/3.57/1000 ms). For anatomical images acquired on the other three scanners, an optimised 3D modified driven equilibrium Fourier transform (MDEFT) sequence was used to acquire 176 sagittal slices with a matrix size of 256 × 224, yielding a final spatial resolution of 1 mm isotropic voxels: repetition time/echo time/inversion time = 12.24/3.56/530 ms and 7.92/2.48/910 ms at 1.5 T and 3 T, respectively (Deichmann et al., 2004).

The T1-weighted anatomical whole-brain volume of each patient was subsequently analysed with our automated lesion identification toolbox using default parameters (for more details, see Seghier et al., 2008). This converts a scanner-sensitive raw image into a quantitative assessment of structural abnormality that should be independent of the scanner used. The procedure combines a modified segmentation-normalisation routine with an outlier detection algorithm according to the fuzzy logic clustering principle (for more details, see Seghier et al., 2007). The outlier detection algorithm assumes that a lesioned brain is an outlier in relation to normal (control) brains. The output includes two 3D lesion images in standard MNI space, generated at a spatial resolution of 2 × 2 × 2 mm^3^. The first is a fuzzy lesion image that encodes the degree of structural abnormality on a continuous scale from 0 (completely normal) to 1 (completely abnormal) at each given voxel relative to normative data drawn from a sample of 64 neurologically-normal controls. A voxel with a high degree of abnormality (i.e. a value near to 1 in the fuzzy lesion image) therefore means that its intensity in the segmented grey and white matter deviated markedly from the normal range. The second is a binary lesion image, which is simply a thresholded (i.e. lesion/no lesion) version of the fuzzy lesion image. All our statistical analyses were based on the fuzzy images. The binary images were used to delineate the lesions, to estimate lesion size and to create lesion overlap maps.

### 2.4 Lesion-deficit analyses

We used voxel-based morphometry (Ashburner and Friston, 2000, Mechelli et al., 2005) to assess lesion-deficit relationships (Mummery et al., 2000, Tyler et al., 2005), performed in SPM12 using the general linear model. The imaging data entered into the voxel-based analysis were the fuzzy (continuous) lesion images that are produced by our automated lesion identification toolbox.

The most important advantage of utilising the fuzzy lesion images (as in Price et al., 2010a) over alternative methods is that they provide a quantitative measure of the degree of structural abnormality, at each and every voxel of the brain, relative to neurologically-normal controls. In contrast to fuzzy lesion images, (i) binary lesion images do not provide a continuous measure of structural abnormality and will be less sensitive to subtle changes that are below an arbitrary threshold for damage (e.g., Fridriksson et al., 2013; Gajardo-Vidal et al., 2018); (ii) normalised T1 images do not distinguish between typical and atypical (abnormal) variability in brain structure (e.g., Stamatakis and Tyler, 2005); and (iii) segmented grey or white matter probability images when used in isolation (as in standard VBM routines) do not provide a complete account of the whole of the lesion (e.g., Mehta et al., 2003).

**Fig. 1:**
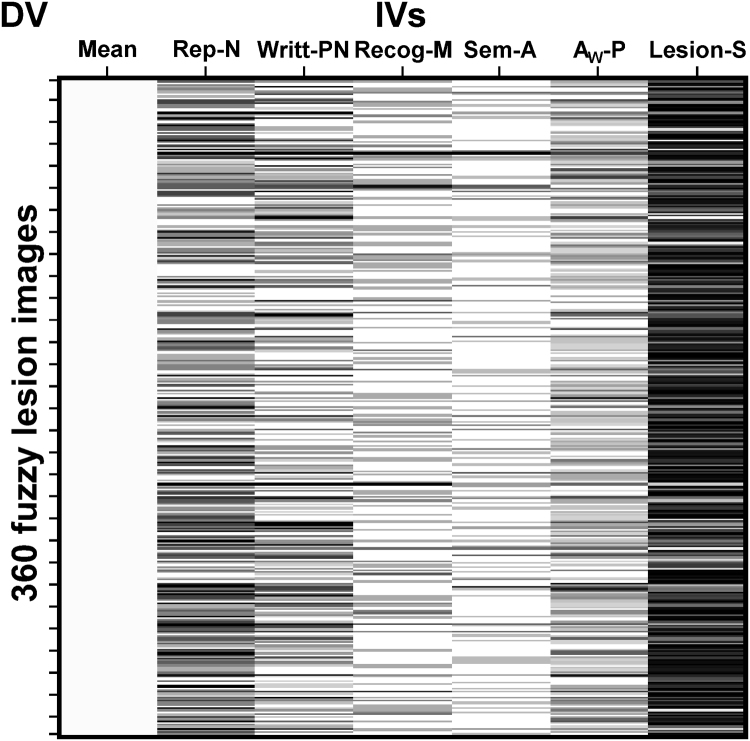
Design matrix. The design matrix for Analysis 1 is shown, where the columns represent the subject-specific independent variables (IVs), with one value for each subject, and the rows correspond to the dependent variable (DV) indexing the degree of structural abnormality in the fuzzy lesion images.

**Fig. 2:**
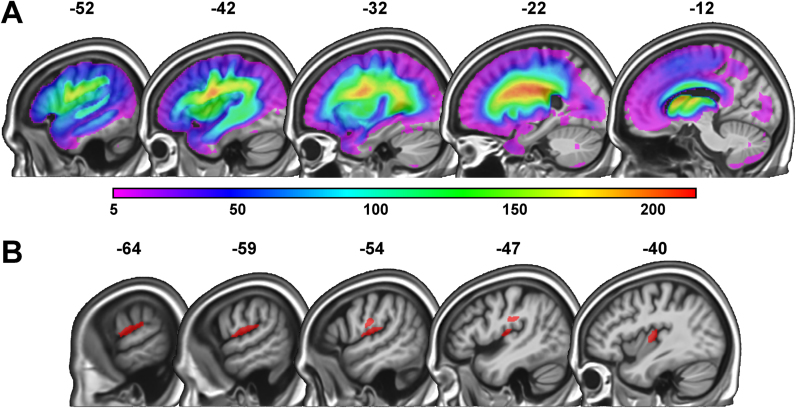
Lesion overlap map and region of interest from Analysis 1. **(A)** Lesion overlap map for the full sample of 360 stroke patients, depicting voxels that were damaged in a minimum of 5 and a maximum of 215 patients. The colour scale indicates the number of patients with overlapping lesions at each given voxel. **(B)** In red, the region of interest identified in Analysis 1 (i.e. 549 voxels) where a significant association between lesion load and speech articulation abilities was found. (For interpretation of the references to color in this figure legend, the reader is referred to the web version of this article.)

In Analysis 1, the fuzzy lesion images were entered into a voxel-based multiple regression model with 6 different regressors (5 behavioural scores and lesion size); see Fig. 1. The regressor of interest was nonword repetition scores that are sensitive to difficulties articulating speech. In addition, the following regressors were included to factor out other sources of variance: written picture naming scores (which are sensitive to name retrieval abilities), semantic associations scores (which are sensitive to visual recognition and semantic processing), auditory word-to-picture matching scores (which are sensitive to auditory recognition and lexical-semantic processing), recognition memory scores (which are sensitive to picture recognition and memory) and lesion size (to partial out linear effects of lesion size). For the voxel-based lesion-deficit analysis (with 360 patients), the search volume was restricted to voxels that were damaged in at least five patients (as in Fridriksson et al., 2016; for rationale, see Sperber and Karnath, 2017). To this end, a lesion overlap map based on the binary lesion images from all 360 patients was created, thresholded at five, and used as an inclusive mask before estimating the model (see Fig. 2A). Our statistical voxel-level threshold was set at *p* < 0.05 after family-wise error (FWE) correction for multiple comparisons (using random field theory as implemented in SPM; Flandin and Friston, 2015) across the whole search volume (for alternative approaches, see Mirman et al., 2018).

Having identified a significant lesion-deficit mapping, we quantified the strength of the association between lesion and deficit by: (i) extracting the raw signal (which indexes the degree of structural abnormality) from each statistically significant voxel; (ii) averaging the signal across voxels (i.e. a single value per patient); and, finally, (iii) computing the partial correlation between lesion load in the region of interest and nonword repetition scores, after adjusting for the effect of the covariates of no interest (i.e. 4 behavioural scores and lesion size). Our measure of effect size was the proportion of variance (= *R*^2^) in nonword repetition scores explained uniquely by lesion load in the region of interest (i.e. the best estimate of the true population effect that we have).

In Analysis 2, we investigated how sample size affected the reproducibility of the lesion-deficit mapping within the region of interest identified in Analysis 1. Specifically, we generated 6000 bootstrap samples of the following sizes: 360, 180, 120, 90, 60 and 30 (i.e. 36,000 resamples in total). These sample sizes were selected to follow as closely as possible those observed in the vast majority of published voxel-based lesion-deficit mapping studies (e.g., Dressing et al., 2018; Fridriksson et al., 2013, Fridriksson et al., 2016; Halai at el, 2017; Schwartz et al., 2011, Schwartz et al., 2012). For each iteration of the resampling procedure, individuals were drawn randomly from the full set of 360 patients with replacement, meaning that the probability of being chosen remained constant throughout the selection process (i.e. the procedure satisfied the Markovian, memory-less, property). For each bootstrap sample, the partial correlation between nonword repetition scores and lesion load (averaged across voxels in the region of interest from Analysis 1) was computed. The resulting *R*^2^ and *p* values were recorded, after regressing out the variance accounted for by the covariates of no interest. Of note, when we re-ran the resampling procedure outlined above with the replacement feature disabled (i.e. sampling without replacement), virtually the same results were obtained (for more details, see Supplementary Material).

In addition, to rule out the possibility that variability in the results could simply be explained by differences in the distribution of damage across the brain, we quantified statistical power in the region of interest (ROI) from Analysis 1 for a representative subset of bootstrap samples. Specifically, only those resamples that produced an *R*^2^ value which fell exactly at a particular decile (i.e. 0th, 10th, 20th…100th) of the distribution of effect sizes were considered. This resulted in the selection of a total of 66 bootstrap samples (i.e. 11 for each sample size); see Table 2. Critically, our power calculations show where in the brain there was sufficient statistical power to detect a significant lesion-deficit association at a threshold of *p* < 0.05 after correction for multiple comparisons. The statistical power maps were generated using the “nii_powermap” function of NiiStat (https://www.nitrc.org/projects/niistat/), which is a set of Matlab scripts for analysing neuroimaging data from clinical populations.

**Table 2:** Statistical power in the region of interest.

**%tile**		**Sample size**					
		**30**	**60**	**90**	**120**	**180**	**360**
**0th**	Power	98%	100%	100%	100%	100%	100%
	*R*^2^	0.00	0.00	0.00	0.00	0.00	0.01
	*P*	0.999	0.999	0.999	0.999	0.404	0.093
**10th**	Power	99%	100%	100%	100%	100%	100%
	*R*^2^	0.01	0.03	0.04	0.05	0.06	0.07
	*P*	0.638	0.218	0.064	0.015	0.001	0.000
**20th**	Power	63%	100%	100%	100%	100%	100%
	*R*^2^	0.03	0.05	0.06	0.07	0.08	0.09
	*P*	0.400	0.093	0.022	0.004	0.000	0.000
**30th**	Power	86%	100%	100%	100%	100%	100%
	*R*^2^	0.06	0.07	0.08	0.08	0.09	0.10
	*P*	0.250	0.046	0.009	0.002	0.000	0.000
**40th**	Power	92%	100%	100%	100%	100%	100%
	*R*^2^	0.08	0.09	0.10	0.10	0.10	0.11
	*P*	0.158	0.025	0.004	0.001	0.000	0.000
**50th**	Power	98%	100%	100%	100%	100%	100%
	*R*^2^	0.11	0.11	0.11	0.11	0.11	0.11
	*P*	0.099	0.012	0.002	0.000	0.000	0.000
**60th**	Power	100%	100%	100%	100%	100%	100%
	*R*^2^	0.15	0.14	0.13	0.13	0.13	0.12
	*P*	0.060	0.006	0.001	0.000	0.000	0.000
**70th**	Power	83%	100%	100%	100%	100%	100%
	*R*^2^	0.18	0.16	0.15	0.14	0.14	0.13
	*P*	0.032	0.002	0.000	0.000	0.000	0.000
**80th**	Power	96%	100%	100%	100%	100%	100%
	*R*^2^	0.23	0.19	0.17	0.16	0.15	0.14
	*P*	0.015	0.001	0.000	0.000	0.000	0.000
**90th**	Power	100%	100%	100%	100%	100%	100%
	*R*^2^	0.30	0.23	0.21	0.19	0.18	0.16
	*P*	0.004	0.000	0.000	0.000	0.000	0.000
**100th**	Power	99%	100%	100%	100%	100%	100%
	*R*^2^	0.79	0.52	0.39	0.39	0.38	0.28
	*P*	0.000	0.000	0.000	0.000	0.000	0.000

The table shows that in all but one case, more than 80% of the voxels comprising the region of interest from Analysis 1 had sufficient statistical power to detect a significant lesion-deficit association at a threshold of *p* < 0.05 after correction for multiple comparisons. %tile = percentile of the effect size (*R*^2^) distribution; Power = percentage of voxels within the region of interest from Analysis 1 that had sufficient statistical power to detect a significant lesion-deficit association at a statistical threshold of *p* < 0.05 after correction for multiple comparisons; *R*^2^ = *R*^2^ value (at a particular decile); *P* = *p* value (at a particular decile).

Importantly, we have chosen to assess in-sample effect sizes, i.e. without validating in a separate data set (Friston, 2012). In this context, the effect size is providing an estimate of the strength of the particular effect identified by our analysis in our data. It may be that an out-of-sample prediction - on new data - would indicate a smaller effect size. However, this would not invalidate the logic of our reasoning, particularly since the essential point we are making here is that our effect size estimate (i.e. approximately 11% in *R*^2^ terms) is very small. If there is inflation in this estimate, it could only mean that the out-of-sample effect size would be even less. Therefore, we have been able to show that even for an over-estimated effect size (if it would turn out to be), there are serious problems that arise from small sample sizes, the fallacy of classical inference, and publication bias. The impact of these issues on the reliability of the findings would only be worse if the effect size were to come down.

Furthermore, we have first statistically selected an ROI in a large sample of patients, with a “left-hemisphere” analysis, and then used smaller and smaller bootstrap samples that focused on the identified ROI. In this sense, we are performing (non-orthogonal) statistical tests in a previously selected ROI, which could potentially inflate false positive rates (Brooks et al., 2017). Consequently, the results derived from the analysis of smaller samples should not be taken as robust findings: they are being presented to make important methodological points. Our best statistical estimates of the effect considered are those obtained from the full data set.

## 3 Results

### 3.1 Analysis 1: identifying a region of interest

Poorer speech articulation was significantly associated with greater lesion load (after controlling for written picture naming, recognition memory, semantic associations and auditory word-to-picture matching scores in addition to lesion size) in 549 voxels (= 4.4 cm^3^ in size; see Table 3). These voxels became our region of interest (ROI) for all subsequent analyses. They were located in parts of the left ventral primary motor and somatosensory cortices (i.e. tongue, larynx, head and face regions), anterior supramarginal gyrus, posterior insula and surrounding white matter (see Fig. 2B).

**Table 3:** Brain regions where lesion load is associated with speech articulation abilities.

**Brain region**	**Peak coordinates**			**Voxel-level**		**Cluster-level**	
	x	y	z	Z-score	*P*_FWE-corr_	Extent	*P*_FWE-corr_
Post-Central	− 60	− 16	12	5.8	0.000	549*	< 0.001
	− 52	− 14	24	4.7	0.009		
	− 56	− 12	18	4.6	0.012		
Posterior Insula	− 40	− 16	8	5.3	0.001		
Anterior SMG	− 66	− 30	20	4.7	0.008		
WM	− 48	− 24	26	4.6	0.010		

The table shows representative (peak) voxels where a significant association between stroke damage and difficulties articulating speech was found. All were in the left hemisphere and the coordinates are reported in MNI space. SMG = supramarginal gyrus; WM = white matter; *P*_FWE-corr_ = *p* value corrected (family-wise error correction) for multiple comparisons.

* At a cluster-forming voxel-wise threshold of *p* < 0.05 FWE-corrected.

**Fig. 3:**
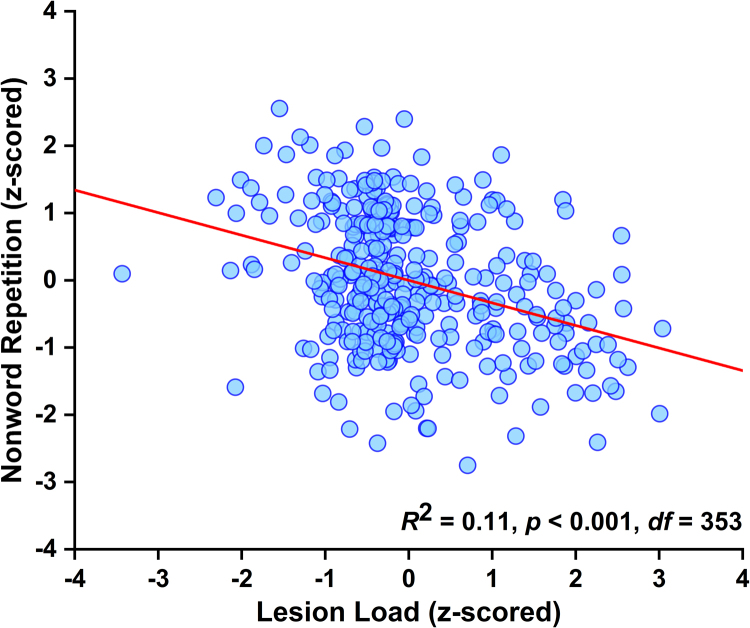
Effect of interest. Visual illustration of the strength of the relationship between lesion load in the region of interest and nonword repetition scores, after factoring out variance explained by the covariates of no interest (i.e. a plot of the lesion load and nonword repetition residuals; Analysis 1).

This highly significant lesion-deficit relationship accounted for 11% of the variance (95% credible interval calculated using a flat prior: 0.06–0.18; Morey et al., 2016); see Fig. 3. In the following analyses, we ask how sample size affects the reproducibility of the identified effect.

### 3.2 Analysis 2: effect size variability and replicability

**Fig. 4:**
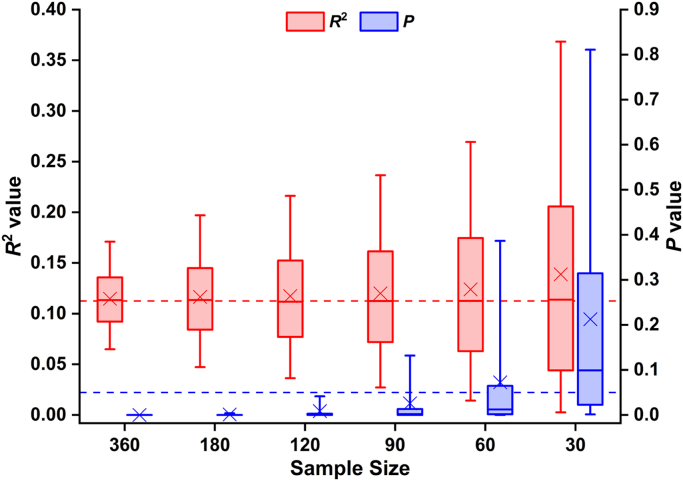
Differential sensitivity of effect sizes and *p* values to sample size. The figure highlights that, while the mean and median of the effect size distributions remained relatively constant across the different sample sizes, the mean and median of the *p* value distributions exhibited substantial and systematic variability. Box plots depict medians with interquartile ranges and whiskers represent the 5th and 95th percentiles. The crosses indicate the mean for each sample size. The horizontal dashed line in red signals the *R*^2^ value obtained in Analysis 1 (including data from all 360 patients), whereas the horizontal dashed line in blue shows the standard alpha level (i.e. 0.05). (For interpretation of the references to color in this figure legend, the reader is referred to the web version of this article.)

**Fig. 5:**
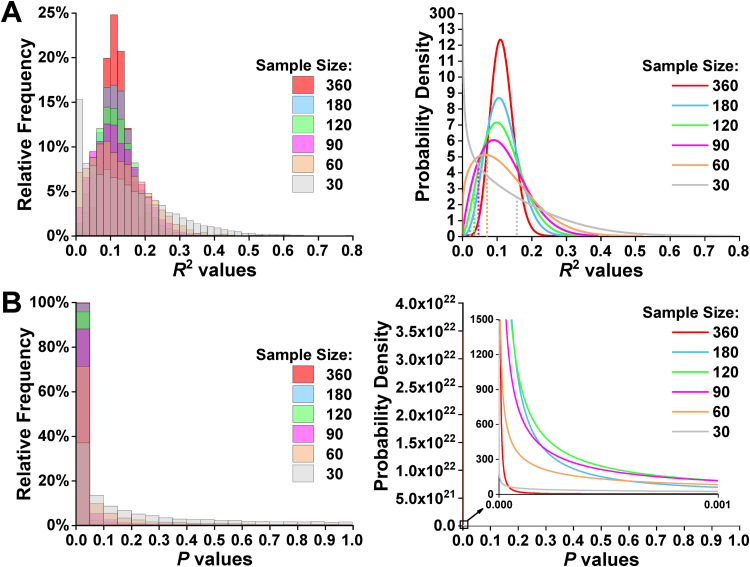
Distribution of *R*^2^ and *p* values. **(A)** From left to right, the frequency (in intervals of 0.02) and probability distributions of effect sizes for each sample size. The vertical dotted lines indicate the boundary between non-significant (*p* ≥ 0.05; to the left) and significant (*p* < 0.05; to the right) *R*^2^ values. **(B)** From left to right, the frequency (in intervals of 0.05) and probability distributions of *p* values for each sample size.

Although the mean/median effect sizes were similar across sample sizes, the mean/median *p* values changed considerably with sample size (see Fig. 4), because there was wide sample-to-sample variability in the extent to which the original effect was replicated. For instance, less than 40% of the random resamples where *N* = 30 generated significant *p* values, while this raised to virtually 100% for the resampled data sets where *N* ≥ 180. Overall, *R*^2^ values ranged between 0.00 and 0.79, whereas *p* values ranged between 6 * 10^–27^ and 1 (see Fig. 5A and B). Additionally, our analyses showed that, as sample size increased, *R*^2^ values tended to fall closer to the mean of the effect size distribution, although a not inconsiderable degree of uncertainty regarding *R*^2^ estimation remained (even for *N* = 180 and 360). In other words, the dispersion of the *R*^2^ values tended to be larger with smaller sample sizes (see Fig. 5A), resulting in less precision in the estimation of the magnitude of the true population effect.

#### 3.2.1 Low-powered resamples can inflate effect sizes

Since studies that obtain statistically non-significant results (i.e. typically *p* ≥ 0.05) are hardly ever published (also known as the file drawer problem or study publication bias), we focused directly upon the resampled data sets that produced significant *p* values. For *N* = 30, the mean and median effect sizes of these significant resamples (i.e. roughly 37%) were 0.26 and 0.24 (range = 0.16–0.79). Conversely, the mean and median effect sizes for the *N* = 30 resamples where the lesion-deficit mapping did not reach statistical significance (i.e. roughly 63%) were 0.07 and 0.06 (range = 0.00–0.16); see Table 4 for similar findings when *N* = 60. Critically, using a more stringent statistical threshold would only aggravate the problem (for more details, see Table 4). With larger sample sizes (*N* ≥ 90), however, effect size inflation is counteracted since both over- and under-estimations of the true effect size surpassed the threshold for statistical significance, resulting in relatively accurate mean estimates (0.13, 0.12, 0.12, and 0.11 respectively).

**Table 4:** Mean and median effect size of the significant and non-significant random data sets by sample size.

***R***^**2**^	**Sample size**											
	**30**		**60**		**90**		**120**		**180**		**360**	
	s	ns	s	ns	s	ns	s	ns	s	ns	s	ns
**Count**	2214	3786	4272	1728	5289	711	5747	253	5974	26	5999	1
	258	5742	1279	4721	2613	3387	3911	2089	5369	631	5997	3
***M***	0.26	0.07	0.16	0.04	0.13	0.03	0.12	0.02	0.12	0.01	0.11	–
	0.45	0.12	0.24	0.09	0.18	0.07	0.15	0.06	0.12	0.05	0.11	0.02
***Mdn***	0.24	0.06	0.15	0.04	0.12	0.03	0.11	0.02	0.11	0.01	0.11	–
	0.43	0.11	0.23	0.09	0.17	0.08	0.14	0.06	0.12	0.05	0.11	0.03
**Min**	0.16	0.00	0.07	0.00	0.05	0.00	0.03	0.00	0.02	0.00	0.03	0.01
	0.38	0.00	0.19	0.00	0.12	0.00	0.09	0.00	0.06	0.00	0.03	0.01
**Max**	0.79	0.16	0.52	0.07	0.39	0.05	0.39	0.03	0.38	0.02	0.28	0.01
	0.79	0.38	0.52	0.19	0.39	0.12	0.39	0.09	0.38	0.06	0.28	0.03

For each summary statistic, the upper row indicates the corresponding value when the alpha threshold was set at 0.05, whereas the lower row indicates the corresponding value when the alpha threshold was set at 0.001. Count = the number of resampled data sets that generated significant or non-significant *R*^2^ values; s = significant (i.e. *p* < α); ns = not significant (i.e. *p* ≥ α); *M* = mean *R*^2^ value; *Mdn *= median *R*^2^ value; Min = minimum *R*^2^ value; Max = maximum *R*^2^ value.

#### 3.2.2 High-powered resamples are sensitive to trivial/small effects

The frequency with which a significant association was observed between lesion load in the ROI and nonword repetition scores increased dramatically with sample size. For example, whereas roughly 37% of the effects for *N* = 30 would be typically regarded as statistically significant (i.e. *p* < 0.05), more than 85% of the lesion-deficit mappings for *N* ≥ 90 generated equally low or even lower *p* values (see Table 4). More importantly, effects as small as 0.05 in *R*^2^ terms (i.e. that only accounted for 5% of the variance) reached statistical significance for *N* = 90; and this phenomenon was even more pronounced in the presence of larger sample sizes: 0.02 for *N* = 180 (see Table 4 and Fig. 5A). Reporting point and interval estimates of effect sizes is therefore essential for assessing the importance or triviality of the identified lesion-deficit mapping, which is particularly relevant when the study uses large sample sizes.

## 4 Discussion

The goal of this study was to examine how sample size influences the reproducibility of voxel-based lesion-deficit mappings. First, we identified a significant lesion-deficit association and estimated its magnitude using data from a very large sample of 360 patients who were all right-handed, English speaking stroke survivors with unilateral left hemisphere damage. By repeating the same analysis on thousands of bootstrap samples of different sizes we illustrate how the estimated effect size, and its statistical significance, varied across replications. This allowed us to index the degree of uncertainty in the estimation of the true population effect as a function of sample size. As expected, effect sizes were more likely to be over-estimated or under-estimated with small sample sizes (i.e. variability in the results increased as sample size decreased). Conversely, we demonstrate how highly significant lesion-deficit mappings can be driven by a negligible proportion of the variance when the sample size is very large.

### 4.1 Estimating the true effect size

The first part of our investigation identified a region of interest (ROI) where damage was reliably associated with impairments in speech articulation. We then calculated what proportion of the variance in nonword repetition scores could be accounted for by the degree of damage to the identified region after factoring out confounds from auditory and visual perception, speech recognition, lexical/semantic processing and word retrieval abilities. The ROI included anatomical brain structures that have been associated with speech production in many previous lesion studies. These include the insula (Ogar et al., 2006), the precentral gyrus, the postcentral gyrus, the supramarginal gyrus and surrounding white matter (Baldo et al., 2011, Basilakos et al., 2015). It did not involve the inferior frontal gyrus/frontal operculum as reported in Hillis et al. (2004) and Baldo et al. (2011), even though our full sample incorporated plenty of patients with damage to these regions (see Fig. 2A). We do not attempt here to adjudicate whether this discrepancy was a consequence of a false negative in our study or a false positive in prior studies. Our focus was on how well the identified lesion-deficit mapping could be replicated across thousands of bootstrap samples drawn randomly from the original data set of 360 patients. For each resample, we estimated how much of the variance in nonword repetition scores could be accounted for by lesion load in the ROI (after adjusting for the effect of the covariates of no interest). These effect sizes and their statistical significance were then compared to our best estimate of the “true” population effect size, which was found (from our full sample of 360 patients) to be 11%.

### 4.2 Variability in the estimated effect size and its statistical significance

The second part of our investigation showed that the probability of finding a significant lesion-deficit association in the ROI from the first analysis (with 360 participants), depended on the size of the sample. For larger samples (*N* ≥ 180), the effect of interest was detected in virtually 100% of resamples. Whereas for smaller samples (*N* = 30), it was detected in less than 40% of resamples (see Table 4). We can also show that *p* values decrease as *N* increases, even when effect sizes are equated (see Fig. 4 and 50th percentile in Table 2). This observation is in line with prior reports that *p* values exhibit wide sample-to-sample variability (Cumming, 2008, Halsey et al., 2015, Vsevolozhskaya et al., 2017), particularly in the presence of small sample sizes (Hentschke and Stuttgen, 2011).

When considering the central tendency of effect size estimates, the difference between larger and smaller resamples is dramatically reduced compared to that seen for *p* values (see mean/median effect sizes in Fig. 4). Nevertheless, even if *p* values were completely abandoned (e.g., Trafimow and Marks, 2015), there is still a great deal of uncertainty in the accuracy with which effect sizes can be estimated when small samples are used. This highlights the importance of reaching a better balance between null-hypothesis significance testing and effect size estimation (Chen et al., 2017, Cumming, 2014, Morey et al., 2014). Indeed, *p* values only indicate the likelihood of observing an effect of a given magnitude (when the null hypothesis is true). As such, they cannot convey the same information provided by point and interval estimates of effect sizes (Steward, 2016, Wasserstein and Lazar, 2016), particularly since the relationship between *p* values and effect sizes is non-linear (Hentschke and Stuttgen, 2011, Simonsohn et al., 2014a, Simonsohn et al., 2014b).

There are several potential reasons why the magnitude and statistical significance of the same effect vary so markedly across resamples. For example, high sample-to-sample variability could reflect (i) sampling error due to heterogeneity in the lesion-deficit association across participants (Button, 2016, Stanley and Spence, 2014), (ii) outliers that are confounding the effects (Rousselet and Pernet, 2012) or (iii) measurement error (Button, 2016, Loken and Gelman, 2017, Stanley and Spence, 2014). In this context, the field needs to adopt informed sampling strategies that ensure representative samples and maximise the probability of identifying generalizable lesion-deficit mappings (Falk et al., 2013, LeWinn et al., 2017, Paus, 2010).

### 4.3 Unreliable effect sizes in smaller samples

High variance in the results of our lesion-deficit mappings with smaller samples (*N* = 30 and 60) demonstrates how effects can be over- as well as under-estimated (e.g., Cremers et al., 2017; Ioannidis, 2008). Indeed, we show that 85% of all significant random data sets for *N* = 30 yielded effect size estimates that were larger than the upper bound of the credible interval (see Table 5). This is consistent with prior observations that low-powered studies (with small sample sizes) can only consistently detect large deviations from the true population effect (Szucs and Ioannidis, 2017). Put another way, even when effect sizes are accurately estimated from small samples, they are unlikely to attain statistical significance; particularly when the magnitude of the effect under investigation is small or medium. In our data, for example, we found that more than half the analyses with *N* = 30 that did not reach statistical significance produced effect sizes that fell within the credible interval (i.e. accurate estimations of effect sizes resulted in false negatives). Even worse, analyses of small sample sizes can invert the direction of the effect (Gelman and Carlin, 2014) as seen in our data where we found that 5% of all results for *N* = 30 were in the wrong direction. Furthermore, reporting such findings as if they were accurate representations of reality would lead to misleading conclusions (Nissen et al., 2016).

**Table 5:** Frequency of accurate and inaccurate effect size estimates by sample size and statistical significance.

***N***	**Effect size**					
	**Significant**			**Not significant**		
	**> 95% CI**	**= 95% CI**	**< 95% CI**	**> 95% CI**	**= 95% CI**	**< 95% CI**
**360**	173	5686	140	0	0	1
**180**	556	4925	493	0	0	26
**120**	795	4430	522	0	0	253
**90**	1081	3887	321	0	0	711
**60**	1417	2855	0	0	421	1307
**30**	1873	341	0	0	2007	1779

The table shows, for each sample size, the frequency with which effect size estimates reached statistical significance (i.e. *p* < 0.05) and fell within (=) or outside the 95% credible interval (i.e. 0.06–0.18) of the best estimate of the “true” population effect (i.e. *R*^2^ = 0.11). 95% CI = 95% credible interval; > = larger than the upper bound of 95% CI; < = smaller than the lower bound of 95% CI.

Critically, the problem was not solved but became worse when we adopted a more stringent statistical threshold, which is contrary to that proposed by Johnson (2013) and Benjamin et al. (2018). For example, if we were to raise the statistical threshold from *p* < 0.05 to *p* < 0.001 for the *N* = 30 resamples, the statistically significant effect sizes would range from 38% to 79% of the variance (compared to 11% in the full sample of 360 patients). Increasing sample size, however, does improve accuracy, with less than 10% of significant *p* values associated with inflated effect sizes when *N* ≥ 180 (see Table 5).

Given that results are more likely to be published if they reach statistical significance than if they do not (i.e. the file drawer problem or study publication bias), our findings highlight three important implications for future lesion-deficit mapping studies. First, low-powered studies (due to small sample sizes) could lead a whole research field to over-estimate the magnitude of the true population effect. Second, power calculations based on inflated effect sizes from studies with small samples will inevitably over-estimate the statistical power associated with small sample sizes (Anderson et al., 2017). Third, although the mean effect size measured over many studies with small sample sizes will eventually converge on the true effect size, in reality, the same study is seldom replicated exactly and null results are only rarely reported. It has therefore been advocated that, contrary to current practices, it is better to carry out a few well-designed high-powered studies than it is to assimilate the results from multiple low-powered studies (Bakker et al., 2012, Higginson and Munafò, 2016). In brief, large scale studies increase the probability that an identified lesion-deficit mapping is correct (Button et al., 2013a, Szucs and Ioannidis, 2017).

### 4.4 Trivial effect sizes in larger samples

Another important observation from the current study is that, when samples are sufficiently large, relatively weak lesion-deficit associations can be deemed statistically significant (i.e. *p* < 0.05). For instance, effects that only accounted for as little as 3% of the variance reached statistical significance when *N* ≥ 120 - an inferential problem known as the fallacy of classical inference (Friston, 2012, Smith and Nichols, 2018). However, our findings are consistent with the view that this issue can be addressed by reporting point and interval estimates of effect sizes (Button et al., 2013b, Lindquist et al., 2013), which allow one to assess the practical significance (as opposed to statistical significance only) of the results. In other words, it can be argued that the fallacy of classical inference is specific to statistical tests (e.g., *t, F* and/or *p* values), leaving effect sizes largely unaffected (Reddan et al., 2017). Furthermore, there are two important advantages of conducting high-powered studies: (i) they greatly attenuate the impact of study publication bias as both over- and under-estimations of the true effect size will surpass the threshold for statistical significance; and (ii) the precision with which the magnitude of the true population effect can be estimated is substantially improved (Lakens and Evers, 2014; see Table 5 and Figs. 4 and 5A). Our study also indicates that, even with sample sizes as large as *N* = 360, a not inconsiderable degree of uncertainty in *R*^2^ estimation remained, which suggests that increasing sample size beyond this *N* will continue to bring benefit.

### 4.5 Study limitations

The focus of the current paper has been on establishing the degree to which the replicability of lesion-deficit mappings is influenced by sample size. To illustrate our points, we have (i) searched for brain regions where damage is significantly related to impairments in articulating speech; (ii) estimated the strength of the identified lesion-deficit association; and, (iii) run the exact same analysis on thousands of samples of varying size. However, we have not attempted to account for all possible sources of inconsistencies in univariate voxel-based lesion-deficit mapping. Nor have we investigated how our results would change if we selected another function of interest (e.g., word retrieval or phonological processing). Indeed, it has already been pointed out that higher-order functions might be associated with smaller effects than lower-level ones (Poldrack et al., 2017, Yarkoni, 2009).

We also acknowledge that there are many different ways of conducting voxel-based lesion-deficit analyses (for more information see de Haan and Karnath, 2018; Karnath et al., 2018; Rorden et al., 2007; Sperber and Karnath, 2018). We have selected one approach, using mass-univariate multiple regression on continuous measures of structural abnormality, behaviour and lesion size. However, we could have used other types of images or other behavioural regressors. For example, several recent studies have adopted dimensionality reduction techniques, such as principal component analysis (PCA), to transform a group of correlated behavioural measures into a smaller number of orthogonal (uncorrelated) factors (e.g., Butler et al., 2014; Corbetta et al., 2015; Mirman et al., 2015a). This PCA approach has made an important contribution to finding coarse-grained explanatory variables (e.g., Halai et al., 2017; Lacey et al., 2017; Mirman et al., 2015b; Ramsey et al., 2017), but some of its limitations are that it: (i) involves an arbitrary criterion for factor extraction; (ii) ignores unexplained variance when selecting a limited number of components; and, (iii) necessitates subjective, a posteriori, interpretation as to what the components might mean based on the factor loadings, which is not typically clear cut. Instead, we propose that a better solution for tackling orthogonality issues is to adopt both a rigorous sampling strategy as well as behavioural measures that offer an optimal sensitivity-specificity balance.

Finally, we have highlighted that the reliance on small-sized samples of patients in the presence of publication bias can undermine the inferential power of univariate voxel-based lesion-deficit analyses. However, we have not attempted to provide guidance on how prospective power calculations - that correct for the various forms of bias present in scientific publications - can be conducted. Nor have we illustrated how the presence of publication and other reporting biases in the lesion-deficit mapping literature, specifically, can be ascertained. The reason simply being that others have already devoted considerable effort to developing tools that identify and deal with problems such as: (i) the excess of statistically significant findings (e.g., Ioannidis and Trikalinos, 2007); (ii) the proportion of false positives (e.g., Gronau et al., 2017); (iii) the presence of publication bias and questionable research practices (e.g., Du et al., 2017; Simonsohn et al., 2014a, Simonsohn et al., 2014b); (iv) errors in the estimation of the direction and/or magnitude of a given effect (e.g., Gelman and Carlin, 2014); and, (v) sample size calculations that take into account the impact of publication bias and uncertainty on the estimation of reported effect sizes (e.g., Anderson et al., 2017). With respect to statistical power, the situation is further complicated by the fact that - in the context of univariate voxel-based lesion-deficit mapping - it not only depends on the size of the sample, the magnitude of the effect under study and the statistical threshold used (Cremers et al., 2017), but also on the distribution of damage across the brain (which is non-uniform; Inoue et al., 2014; Kimberg et al., 2007; Mah et al., 2014; Sperber and Karnath, 2017). More research on the topic will be required before prospective power calculations can be fully trusted. Until that moment, the recruitment of representative patient samples in combination with high-powered designs seems to be the best available solution to the issues discussed here.

### 4.6 Interpreting voxel-based lesion-deficit mappings

The strength of the lesion-deficit association that we identified in a large sample of 360 patients illustrates that the majority of the variability in speech articulation abilities was driven by factors other than the degree of damage to the ROI. A clear implication of this is that the field of lesion-deficit mapping still has a long way to go before it can inform current clinical practice, which is arguably one of its most important goals. Future studies will need to control and understand other known sources of variance (apart from lesion site and size) such as time post-stroke, age and education in order to improve our ability to predict language outcome and recovery after stroke at the individual patient level (Price et al., 2017). Furthermore, to map all the possible ways in which brain damage can affect behaviour, it will in all likelihood be necessary to use increasingly larger samples of patients (e.g., Price et al., 2010b; Seghier et al., 2016) and multivariate methods (e.g., Hope et al., 2015; Pustina et al., 2018; Yourganov et al., 2016; Zhang et al., 2014).

## 5 Conclusions

This study investigated the impact of sample size on the reproducibility of voxel-based lesion-deficit mappings. We showed that: (i) highly significant lesion-deficit associations can be driven by a relatively small proportion of the variance; (ii) the exact same lesion-deficit mapping can vary widely from sample to sample, even when analyses and behavioural assessments are held constant; (iii) the combination of publication bias and low statistical power can severely affect the reliability of voxel-based lesion-deficit mappings; and, finally, (iv) reporting effect size estimates is essential for assessing the importance or triviality of statistically significant findings. Solutions to the issues highlighted here will, in our view, likely involve the use of: (a) improved reporting standards; (b) increasingly larger samples of patients; (c) multivariate methods; (d) informed sampling strategies; and, (e) independent replications. Careful reflection on some deeply-rooted research practices, such as biases in favour of statistically significant findings and against null results, might also be necessary.
