# Supplementary material for: The impact of sample size on the reproducibility of voxel-based lesion-deficit mappings
Source: Neuropsychologia. 2018 Jul 1;115:101–11. doi: 10.1016/j.neuropsychologia.2018.03.014 (PMC6018568; doi:10.1016/j.neuropsychologia.2018.03.014)

**The impact of sample size on the reproducibility of voxel-based lesion-deficit mappings**

Diego L. Lorca-Puls, Andrea Gajardo-Vidal, Jitrachote White, Mohamed L. Seghier, Alexander P. Leff, David W. Green, Jenny T. Crinion, Philipp Ludersdorfer, Thomas­ M. H. Hope, Howard Bowman, and Cathy J. Price

**Supplementary material**

The supplementary material included here shows that our results are essentially independent of whether the resampling procedure is performed with (see main text) or without (see Tables S1/S2 and Figs. S1/S2) replacement. One minor difference is that the bootstrapping approach (i.e. sampling with replacement) was associated with slightly higher spread of the estimates. Critically, however, this observation does not affect any of the conclusions drawn in the paper.

Additionally, in Monte-Carlo simulations, we compared the “direct” sampling with replacement approach used in the main body of this paper, with a procedure that we call ”indirect” sampling with replacement, and which involves two stages. First, we directly sample, without replacement, a single subsample of size *N*, from the full data set (of 360 patients). We call this size *N* subsample, the base-sample. Then, second, we sample with replacement from the base-sample, generating subsamples that are also of size *N*. The different sample sizes explored in our paper involves *N* systematically reducing in size from 180 to 120 to 90 to 60 to 30.

The aforementioned procedure is intended to address a potential criticism of the other resampling approaches we examine (i.e. “direct” sampling with and without replacement). That is, in all the other approaches, the number of data points shared between different subsamples tends to reduce as the subsample size, *N*, becomes smaller. Such a feature could be seen as increasing the difference between subsamples and thus, the variability across subsamples. The “indirect” sampling scheme avoids this difficulty, since, in the second step, the generated subsamples are always the same size as the sample they are drawn from, i.e. as the base-sample. Our Monte-Carlo simulations showed that the “indirect” sampling with replacement procedure yielded essentially the same results as the other resampling approaches considered here.

**Table S1:** Mean and median effect size of the significant and non-significant random data sets by sample size.

| ***R*^2^** | **Sample Size** | | | | | | | | | |
| --- | --- | --- | --- | --- | --- | --- | --- | --- | --- | --- |
|  | **30** | | **60** | | **90** | | **120** | | **180** | |
|  | s | ns | s | ns | s | ns | s | ns | s | ns |
| **Count** | 2188 | 3812 | 4394 | 1606 | 5473 | 527 | 5912 | 88 | 5999 | 1 |
|  | 217 | 5783 | 1207 | 4793 | 2667 | 3333 | 4088 | 1912 | 5786 | 214 |
| ***M*** | 0.26 | 0.07 | 0.16 | 0.04 | 0.13 | 0.03 | 0.12 | 0.02 | 0.11 | --- |
|  | 0.44 | 0.13 | 0.24 | 0.10 | 0.17 | 0.08 | 0.14 | 0.07 | 0.12 | 0.05 |
| ***Mdn*** | 0.24 | 0.06 | 0.14 | 0.04 | 0.12 | 0.03 | 0.11 | 0.03 | 0.11 | --- |
|  | 0.43 | 0.11 | 0.23 | 0.10 | 0.16 | 0.08 | 0.13 | 0.07 | 0.11 | 0.05 |
| **Min** | 0.16 | 0.00 | 0.07 | 0.00 | 0.05 | 0.00 | 0.03 | 0.01 | 0.03 | 0.02 |
|  | 0.38 | 0.00 | 0.19 | 0.00 | 0.12 | 0.00 | 0.09 | 0.01 | 0.06 | 0.02 |
| **Max** | 0.62 | 0.16 | 0.52 | 0.07 | 0.37 | 0.05 | 0.30 | 0.03 | 0.23 | 0.02 |
|  | 0.62 | 0.38 | 0.52 | 0.19 | 0.37 | 0.12 | 0.30 | 0.09 | 0.23 | 0.06 |

For each summary statistic, the upper row indicates the corresponding value when the alpha threshold was set at 0.05, whereas the lower row indicates the corresponding value when the alpha threshold was set at 0.001. Count = the number of resampled data sets that generated significant or non-significant *R*^2^ values; s = significant (i.e. *p* < α); ns = not significant (i.e. *p* ≥ α); *M* = mean *R*^2^ value; *Mdn* = median *R*^2^ value; Min = minimum *R*^2^ value; Max = maximum *R*^2^ value.

**Table S2:** Frequency of accurate and inaccurate effect size estimates by sample size and statistical significance.

| ***N*** | **Effect Size** | | | | | |
| --- | --- | --- | --- | --- | --- | --- |
|  | **Significant** | | | **Not significant** | | |
|  | **> 95% CI** | **= 95% CI** | **< 95% CI** | **> 95% CI** | **= 95% CI** | **< 95% CI** |
| **180** | 171 | 5680 | 148 | 0 | 0 | 1 |
| **120** | 553 | 4938 | 421 | 0 | 0 | 88 |
| **90** | 900 | 4303 | 270 | 0 | 0 | 527 |
| **60** | 1336 | 3058 | 0 | 0 | 426 | 1180 |
| **30** | 1847 | 341 | 0 | 0 | 2057 | 1755 |

The table shows, for each sample size, the frequency with which effect size estimates reached statistical significance (i.e. *p* < 0.05) and fell within (=) or outside the 95% credible interval (i.e. 0.06-0.18) of the best estimate of the “true” population effect (i.e. *R*^2^ = 0.11). 95% CI = 95% credible interval; > = larger than the upper bound of 95% CI; < = smaller than the lower bound of 95% CI.


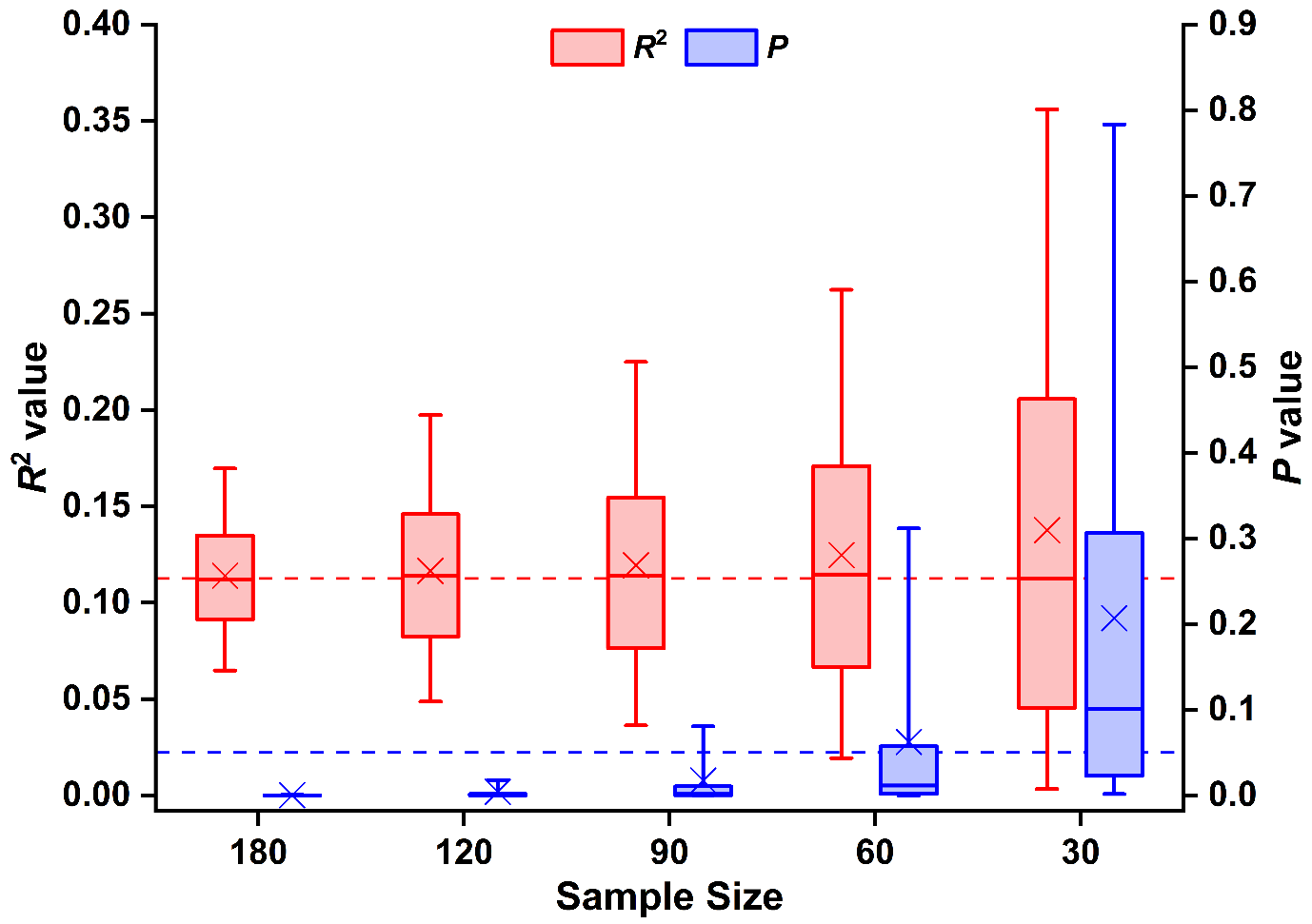


**Fig. S1.** Differential sensitivity of effect sizes and *p* values to sample size. The figure highlights that, while the mean and median of the effect size distributions remained relatively constant across the different sample sizes, the mean and median of the *p* value distributions exhibited substantial and systematic variability. Box plots depict medians with interquartile ranges and whiskers represent the 5th and 95th percentiles. The crosses indicate the mean for each sample size. The horizontal dashed line in red signals the *R*^2^ value obtained in Analysis 1 (including data from all 360 patients), whereas the horizontal dashed line in blue shows the standard alpha level (i.e. 0.05).

**Fig. S2.** Distribution of *R*^2^ and *p* values. **(A)** From left to right, the frequency (in intervals of 0.02) and probability distributions of effect sizes for each sample size. The vertical dotted lines indicate the boundary between non-significant (*p* ≥ 0.05; to the left) and significant (*p* < 0.05; to the right) *R*^2^ values. **(B)** From left to right, the frequency (in intervals of 0.05) and probability distributions of *p* values for each sample size.
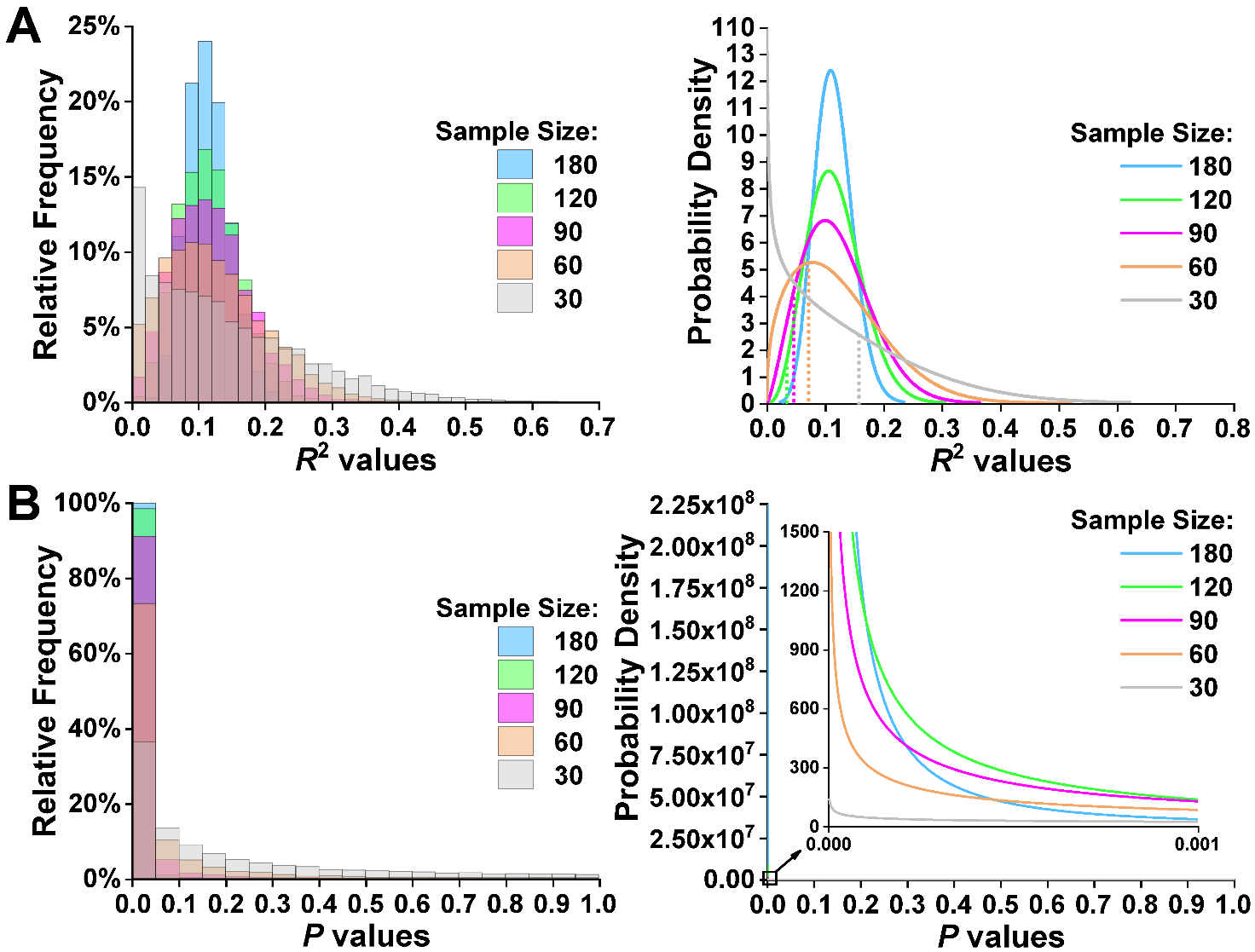

Supplement: Supplementary file 1 — Supplementary material. [file mmc1.docx]
